# Barriers to and facilitators of linkage to care following hypertension and diabetes screening among health workers in Zimbabwe: A mixed method study

**DOI:** 10.1371/journal.pgph.0004513

**Published:** 2025-04-29

**Authors:** Leyla Larsson, Rudo M.S. Chingono, Claire J. Calderwood, Farirai P. Nzvere, Edson T. Marambire, Fungai Kavenga, Sibusisiwe Sibanda, Bridget Kanengoni, Nicol Redzo, Victoria Simms, Chiratidzo E. Ndhlovu, Hilda Mujuru, Simbarashe Rusakaniko, Rashida A. Ferrand, Kalpana Sabapathy, Katharina Kranzer

**Affiliations:** 1 Institute of Infectious Diseases and Tropical Medicine, LMU University Hospital, LMU Munich, Munich, Germany; 2 The Health Research Unit Zimbabwe, Biomedical Research and Training Institute, Harare, Zimbabwe; 3 Department of Clinical Research, London School of Hygiene & Tropical Medicine, London, United Kingdom; 4 Department of Infectious Disease Epidemiology, London School of Hygiene & Tropical Medicine, London, United Kingdom; 5 Internal Medicine Unit, Faculty of Medicine and Health Sciences, University of Zimbabwe, Harare, Zimbabwe; 6 Department of Paediatrics and Child Health, Faculty of Medicine and Health Sciences, University of Zimbabwe, Harare, Zimbabwe; 7 Family Medicine, Global and Public Health Unit, Faculty of Medicine and Health Sciences, Harare, Zimbabwe; University of the Witwatersrand Johannesburg Faculty of Health Sciences, SOUTH AFRICA

## Abstract

The benefits of screening for any condition are only realised if individuals who screen positive link to care services. We investigated linkage to hypertension and diabetes care by healthcare workers accessing a comprehensive health check service. We also explored facilitators and barriers to linkage to care. Between July 2020 and June 2022, a health check with referral and follow-up was offered to healthcare workers (clients) in Zimbabwe. We aimed to understand the proportion that linked to care after referral for an elevated blood pressure and/or HbA1c, assessed by follow-up phone calls. Linkage to care was defined as self-report of having seen a health professional within 30–60 days of the positive screening test result. In-depth interviews were conducted with 15 clients to understand associated facilitators and barriers. Overall, 3,143 clients accessed screening services. The majority were women (75.7%), and median age was 37 (IQR: 28–46) years. 785 (25.0%) clients screened positive for hypertension and 279 (8.9%) screened positive for diabetes. Clients referred for diabetes were more likely to accept referral (n=212, 72.0%) than those referred for hypertension (n=323, 41.1%). Among those referred and successfully contacted for follow-up, 131/182 (72.0%) reported having linked to care for diabetes and 218/269 (81.0%) for hypertension. Distance, accessibility, and travel costs to the facility they were referred to, influenced the decision and ability to link to care. While linkage to care for hypertension and diabetes was high among those who accepted referral, many healthcare workers did not accept referral. Greater awareness among healthcare providers regarding the importance of NCD care to improve acceptance of referral is required and every step of the care cascade must be affordable, accessible, and patient-centred.

## Introduction

Noncommunicable diseases (NCDs) account for 74% of deaths among adults globally, amounting to over 41 million deaths each year [1]. Most of the NCD burden (77%) is concentrated in low- and middle-income countries (LMICs) [1]. In Southern Africa, one in two adults with diabetes and two in three with hypertension remain undiagnosed [2,3]. Considering both conditions are relatively easy to diagnose and treatable with cost-effective and efficacious interventions, there is a missed opportunity to reduce the burden of NCD-related complications in the region [4,5].

We have previously reported high prevalence of NCDs among healthcare workers in Zimbabwe [6]. While diagnosis is the first step in the continuum of care, preventing NCD-associated morbidity and mortality requires that people are able to access treatment and remain in care. Linkage to care for NCDs is a challenge in resource-limited settings; barriers to NCD care include health systems designed for acute disease management, incomplete programme implementation, low individual perceived risk of complications, poor patient-provider relationships, suboptimal and complex referral systems, and affordability [7–10].

Zimbabwe’s health system was once considered among the best in Africa but, as a result of lack of public health financing (investment into health financing has declined by 7.6% between 2010 and 2016), poor working conditions and poor renumeration of healthcare workers, and frequent stockouts of essential commodities, it is now severely challenged. Since the free public-sector healthcare of the 1980s and 1990s, [11–13] patient-level clinic access fees have been introduced and medications, except for a few select conditions such as HIV and tuberculosis, incur out of pocket costs [14]. This in turn has led to Zimbabwe having one of the highest rates of private health insurance globally [15]. Healthcare costs covered by private providers depend on the premium and the insurance plan. Over the past six years, Zimbabwean public sector health workers have experienced substantial real-terms pay cuts due to crippling inflation rates ranging between 10.6% in 2018 and 557.2% in 2020 (for 2022 the inflation rate was 104.7%) [16]. This has contributed to out-migration of health professionals and industrial action resulting in further deterioration of the healthcare system [17–19].

With this challenging context in mind, this study aimed to evaluate whether health workers who accessed a comprehensive health check and were found to have elevated blood pressure and/or glycosylated haemoglobin A1c (HbA1c) were able to access care and treatment; as well as exploring facilitators and barriers to linkage to care for these conditions.

## Methods

This study followed-up people who screened positive for elevated blood pressure (hypertension) and/or HbA1c (diabetes) as part of a comprehensive health check offered to health workers, henceforth referred to as clients, in Zimbabwe, as described in detail elsewhere [6,20,21]. Briefly, from 29 July 2020–31 July 2022 a comprehensive health check, including testing for diabetes and hypertension, was offered to clinical and non-clinical health workers in primary, secondary, and tertiary healthcare facilities from seven out of ten provinces in Zimbabwe. Clients were offered referrals if screening results suggested an undiagnosed condition or if the condition was previously diagnosed but uncontrolled. Following feedback from ongoing synchronous process evaluation, follow-up phone calls to facilitate linkage to care were implemented from December 2020.

Blood pressure was measured three times using an automated blood pressure machine, with the client seated and at intervals of at least five minutes. The lowest measurement was used as the client’s final blood pressure to inform discussions about lifestyle changes and referral for onward care. An elevated blood pressure was defined using World Health Organization (WHO) criteria as systolic blood pressure (sBP) ≥140mmHg and/or a diastolic blood pressure (dBP) ≥90mmHg [22]. Service providers offered referrals for clients meeting criteria for hypertension. Hypertension with sBP ≥180mmHg and/or dBP ≥120mmHg and pregnant women with elevated blood pressure were referred to the emergency department [23]. For diabetes screening, a point-of-care HbA1c test (SD Biosensor A1c Care [SD Biosensor, Singapore]) was performed and a client was referred if the HbA1c result ≥6.5% [24]. Nutritional status was additionally measured using body mass index (BMI) and categorised into underweight (<18.5 kg/m^2^), normal (18.5-24.9 kg/m^2^), overweight (25-29.9 kg/m^2^), and obese (≥30 kg/m^2^).

**Fig 1:**
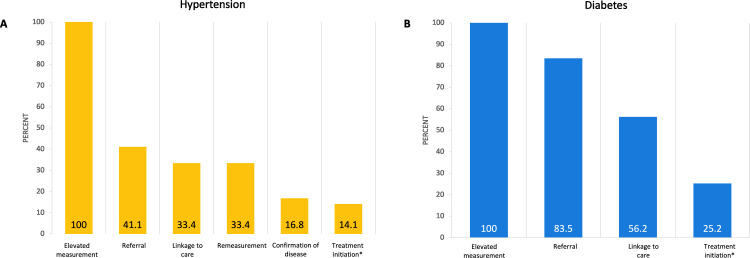
Hypertension (A) and diabetes (B) care cascade after elevated result at health screening. The denominator at each step is the number from the previous step. An assumption was made that the linkage proportion was the same among those who were not successfully contacted as for those who were contacted. N = 785 for hypertension and N = 279 for diabetes. A detailed breakdown including absolute numbers at each step per condition is found in Fig 3. Both people newly screening positive and those who had known disease but had an elevated result at the screening visit (i.e., uncontrolled disease) were eligible for referral and included in the figure. *Treatment initiation includes both new treatment initiation and those who were already on treatment and those reviewed with changes to their treatment regimen or treatment continuation.

In this study, linkage to care is framed in a care cascade and ascertained into discrete steps, notably splitting the linkage to care steps into referral and seeking further care (Fig 1). For hypertension, the care cascade consisted of i) positive screen, ii) referral, iii) linkage to care, iv) remeasurement, v) confirmation of disease, and vi) treatment initiation, modification, or continuation (Fig 1A). For diabetes, the remeasurement and confirmation of disease steps are removed from the cascade as this information was not collected (Fig 1B).

### Referral process

Clients meeting referral criteria and not already in care were offered referral to a provider of their choice, including primary health care facilities, private general practitioners and outpatient clinics in hospital. Those who accepted referral were provided with standardised referral letters including all screening results. Those with a known diagnosis and already in care were also offered the referral letter to give to their treating clinician. For people with positive hypertension screen, the referral letter recommended repeat blood pressure measurement and treatment to be offered if hypertension was confirmed. For people with positive diabetes screen, the referral letter recommended repeat assessment and treatment at provider’s discretion. A client was categorised to as having linked to care if they reported that they had seen a health professional following the positive screen from the health check.

### Ascertainment of linkage to care

Clients who were referred were asked to provide their contact details and written informed consent to receive follow-up phone calls to facilitate and establish linkage to care. More than one phone number was obtained for each client and service providers were asked to call the phone number at the time of referral to ensure it was correct. Clients who were referred for any condition were called between 30–60 days after accessing the service. Also, clients who had not linked to care at the time of the first follow-up contact were called for a second follow-up three to six months after the health check.

Three attempts were made to establish contact. A short telephone-based questionnaire was administered inquiring about contact with health providers since attending the health check. For people who had linked to care, details of repeat blood pressure measurements and start or modification of treatment for diabetes or hypertension were collected; for those who did not link to care, questions were asked about reasons for not linking to care.

### Data collection and analysis

Data collection in this study followed a sequential mixed methods design. Quantitative data was first collected. This was followed by qualitative data collection through semi-structured interviews with a subset of survey participants. The survey data informed the development of the interview guide, allowing us to explore the quantitative findings in greater depth.

### Quantitative analysis

At the time of screening, electronic case report forms captured data on socio-demographics, past medical history, uptake of screening, results, and whether a person was referred, using SurveyCTO software, loaded onto electronic tablets [25]. Information about follow-up and linkage to care was recorded on paper forms at the time of the phone call and entered into the electronic database. Data were stored on a server at the Biomedical Research and Training Institute (BRTI). Any identifying information was held separately, on paper, in locked cabinets.

This analysis included data from clients accessing the service between July 1^st^ 2021 and July 18^th^ 2022, reflecting the period during which first follow-up calls were systematically implemented at 30–60 days. The primary outcome of interest was self-reported linkage to care after a health check with a positive hypertension or diabetes screen at the first or second follow-up call. The analysis was conducted for each condition separately and stratified by whether the client was newly diagnosed or previously diagnosed with the condition, but the condition not controlled.

Data was analysed and visualised using R version 4.1.3. Baseline characteristics of the study population were assessed using proportions for categorical variables and medians with interquartile ranges (IQR) or means with standard deviations (SD) for continuous variables. Statistical significance using t-tests was used to assess the differences between blood pressure measurements among those who accepted referrals and those who did not accept referral.

### Qualitative analysis

In-depth interviews were conducted, using a phenomenological approach, with clients referred for care and service providers conducting the health checks to understand i) the facilitators and barriers to linkage to care and ii) possible interventions to improve linkage to care. For this, a topic guide was developed and clients that had either linked to care or not yet linked to care (for participant characteristics see S1 Table) were purposively sampled with an attempt to reduce selection bias. Clients were selected from a range of facilities, balanced by sex, with various health conditions, and a mixture of those who linked to care or failed to link to care. They were informed about the purpose of the interview, which was to understand their experiences with linking to care. Selection was done until saturation was reached. Data saturation was reached when no new information was found, whereby the researcher was not able to derive new categories, sub-themes, or themes [26]. The service providers that screened clients were asked to provide informal feedback on the referral process to understand why clients would or would not take up referrals. All interviews were conducted face-to-face, at the health facilities, by a trained qualitative research assistant (SS), in English or the local language (Shona or Ndebele) preferred by the client. The interviews were audio-recorded, transcribed verbatim, and those conducted in local languages were translated into English. The transcripts were not member-checked due to logistical constraints but the transcripts went through thorough peer debriefing and preliminary findings were presented to a small group of hospital stakeholders. Data were analysed by a qualitative research assistant (SS) and a social scientist (RC) using the six steps in thematic analysis [27]. The process started with data familiarisation, with the two researchers independently inductively coding the first four transcripts. The coding was then compared between the two of them, differences were discussed, and a coding framework was generated where categories, sub-themes, and themes were defined and named. The remaining transcripts were deductively coded. The analysis was conducted in NVivo 14 [28].

### Ethics

Ethical approval was granted by the Institutional Review Board at BRTI, the Medical Research Council of Zimbabwe (MRCZ/A/2627) and the London School of Hygiene & Tropical Medicine (LSHTM) ethics committee (22514). Permission was obtained from medical directorates to operate in health facilities of their jurisdiction. The study requested, and was granted, a waiver allowing for verbal rather than written consent by clients accessing the service, since the primary aim of the project was to provide a service. Written informed consent was obtained for follow-up phone calls and for all qualitative interviews.

## Results

### Screening and acceptance of referral to care

Between July 1^st^ 2021 and July 18^th^ 2022, 3,143 clients accessed the health check. The majority were women (n=2,379, 75.7%) and the median age was 37 (IQR: 28–46) years (Table 1). Most (n=2,423, 77.1%) worked at a hospital, 717 (22.8%) worked at clinics, and the remainder worked for non-governmental organisations or health trusts. Two thirds of clients (n=2,023, 64.4%) were obese or overweight. 2,019/3,143 (64.2%) clients reported having medical aid.

**Table 1:** Client characteristics stratified by high blood pressure and HbA1c and whether they accepted referral.

		Hypertension			Diabetes		
	Clients accessing the service (n=3,143)	**Positive screen (n=785)**	Accepted referral (n=323)	Did not accept referral (n=462)	**Positive screen (n=279)**	Accepted referral (n=233)	Did not accept referral (n=46)
**Sex**							
Female	2,379	**586**	238 (40.6%)	348 (59.4%)	**217**	176 (81.1%)	41 (18.9%)
Male	764	**199**	85 (42.7%)	114 (57.3%)	**62**	57 (91.9%)	5 (8.1%)
**Age**							
< 30	955	**86**	20 (23.3%)	66 (76.7%)	**21**	15 (71.4%)	6 (28.6%)
31 – 40	942	**208**	86 (41.3%)	122 (58.7%)	**66**	55 (83.3%)	11 (16.7%)
> 40	1,246	**491**	217 (44.2%)	274 (55.8%)	**192**	163 (84.9%)	29 (15.1%)
**Occupation**							
Clinical	1,814	**415**	172 (41.4%)	243 (58.6%)	**158**	126 (79.7%)	32 (20.3%)
Non-clinical	1,329	**370**	151 (40.8%)	219 (59.2%)	**121**	107 (88.4%)	14 (11.6%)
**Highest education**							
O-level	1,316	**374**	167 (44.7%)	207 (55.3%)	**140**	112 (80.0%)	18 (20.0%)
A-level	367	**36**	13 (36.1%)	23 (63.9%)	**13**	8 (61.5%)	5 (38.5%)
Post-secondary	1,082	**287**	116 (40.4%)	171 (59.6%)	**110**	94 (85.5%)	16 (14.5%)
University	378	**88**	27 (30.7%)	61 (69.3%)	**26**	19 (73.1%)	7 (26.9%)
**Medical aid**							
No	1,124	**224**	92 (41.1%)	132 (58.9%)	**69**	14 (20.3%)	55 (79.7%)
Yes	2,019	**561**	231 (41.2%)	330 (58.8%)	**210**	41 (19.5%)	169 (80.5%)
BMI*							
Underweight	81	**10**	3 (30.0%)	7 (70.0%)	**2**	2 (100.0%)	0 (0.0%)
Normal	1,031	**169**	63 (37.3%)	106 (62.7%)	**43**	35 (81.4%)	8 (18.6%)
Overweight	946	**232**	98 (42.2%)	134 (57.8%)	**90**	76 (84.4%)	14 (15.6%)
Obese	1,077	**373**	159 (42.6%)	214 (57.4%)	**142**	118 (83.1%)	24 (16.9%)
HIV status**							
Positive	376	**107**	49 (45.8%)	58 (54.2%)	**41**	33 (80.5%)	8 (19.5%)
Negative	1,542	**375**	143 (38.1%)	232 (61.9%)	**131**	107 (81.7%)	24 (18.3%)
Unknown	1,225	**303**	131 (43.2%)	172 (56.8%)	**107**	93 (86.9%)	14 (13.1%)

***Footnotes:*** *Eligibility for referral was defined as being above the cut-off measurement for either blood pressure (sBP > 140mmHg and/or dBP) > 90mmHg) or HbA1c ≥ 6.5%. Clients could have both elevated blood pressure and elevated HbA1c.*

*8 clients are missing height and weight measurements

** HIV status considers reported HIV status as well as the on-site HIV test results (using blood-based testing or self-testing using oral mucosal transudate tests)

***Abbreviations:*** *body mass index (BMI)*

***Definitions*** *for BMI, underweight is defined as having a BMI < 18.5 kg/m*^*2*^*, normal as a 18.5 ≤ BMI < 25 kg/m*^*2*^*, overweight as a 25 ≤ BMI < 30 kg/m*^*2*^*, and obese as a BMI ≥ 30 kg/m*^*2*^*. For education, O-level marks eleven years of education and the end of the secondary education cycle and A-level adds an additional two years of education (total 13 years) to receive a pre-university qualification degree.*

A total of 785/3,143 (25%) clients screened positive for hypertension, of which 475/785 (60.5%) were undiagnosed and 310/785 (39.5%) were previously diagnosed. Two hundred and seventy nine (8.9%) had a positive diabetes screen, of which 223/279 (79.9%) were undiagnosed and 56/279 (20.1%) were previously diagnosed (Table 1). Overall, 114/3,143 (3.6%) clients had a positive screen for both conditions; among these 46/114 (40.4%) did not have a previous diagnosis of either hypertension or diabetes, 39/114 (34.2%) already knew they had hypertension, 9/114 (7.9%) knew they had diabetes and 20/114 (17.5%) knew they had both conditions. Among the 53 clients with previously diagnosed diabetes and elevated HbA1c, the median HbA1c was 8.7% (IQR: 7.1-10.0) and among the 310 clients with previously diagnosed hypertension, the median systolic (sBP) and diastolic (dBP) blood pressures were 145 (IQR: 135–157) and 96 (IQR: 91–103) respectively.

**Fig 2:**
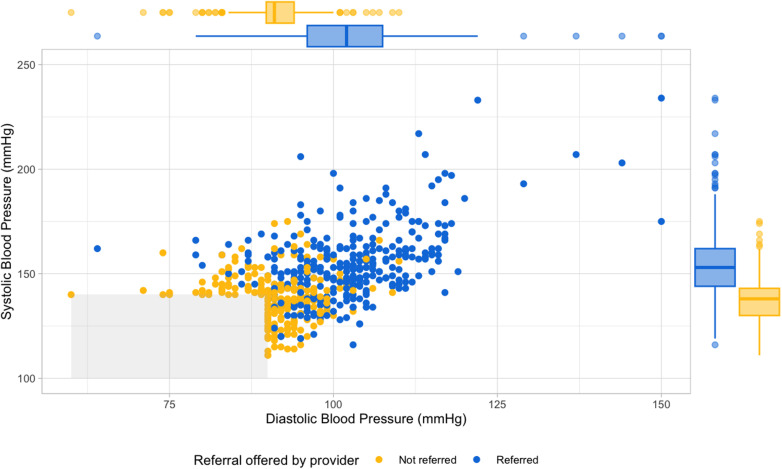
Systolic and diastolic blood pressure by whether a client was offered referral by a provider. Scatterplot of clients with elevated blood pressure (n=547) stratified by whether they were offered referral (blue = offered referral [n=323; 59%] and yellow = not offered referral [224; 41%]). The grey-shaded box is blood pressure results which were not eligible for referral (i.e., systolic blood pressure < 140mmHg and diastolic blood pressure < 90mmHg). Colour-coordinated boxplots show the median and associated interquartile ranges (IQR) of the systolic blood pressure (vertical; offered referral = median: 153mmHg, IQR: 144–162mmHg; not offered referral = median: 138mmHg, IQR: 130–143mmHg) and the diastolic blood pressure (horizontal; offered referral = median 102mmHg and IQR: 96–108mmHg; not offered referral = median 91mmHg and IQR 90–94mmHg).

Fig 2 shows the distribution of systolic and diastolic blood pressure stratified by whether people were offered referral or not. Mean sBP and dBP was higher among those who were offered referral compared to those who were not (p < 0.001 for both sBP and dBP; Fig 2).

**Fig 3:**
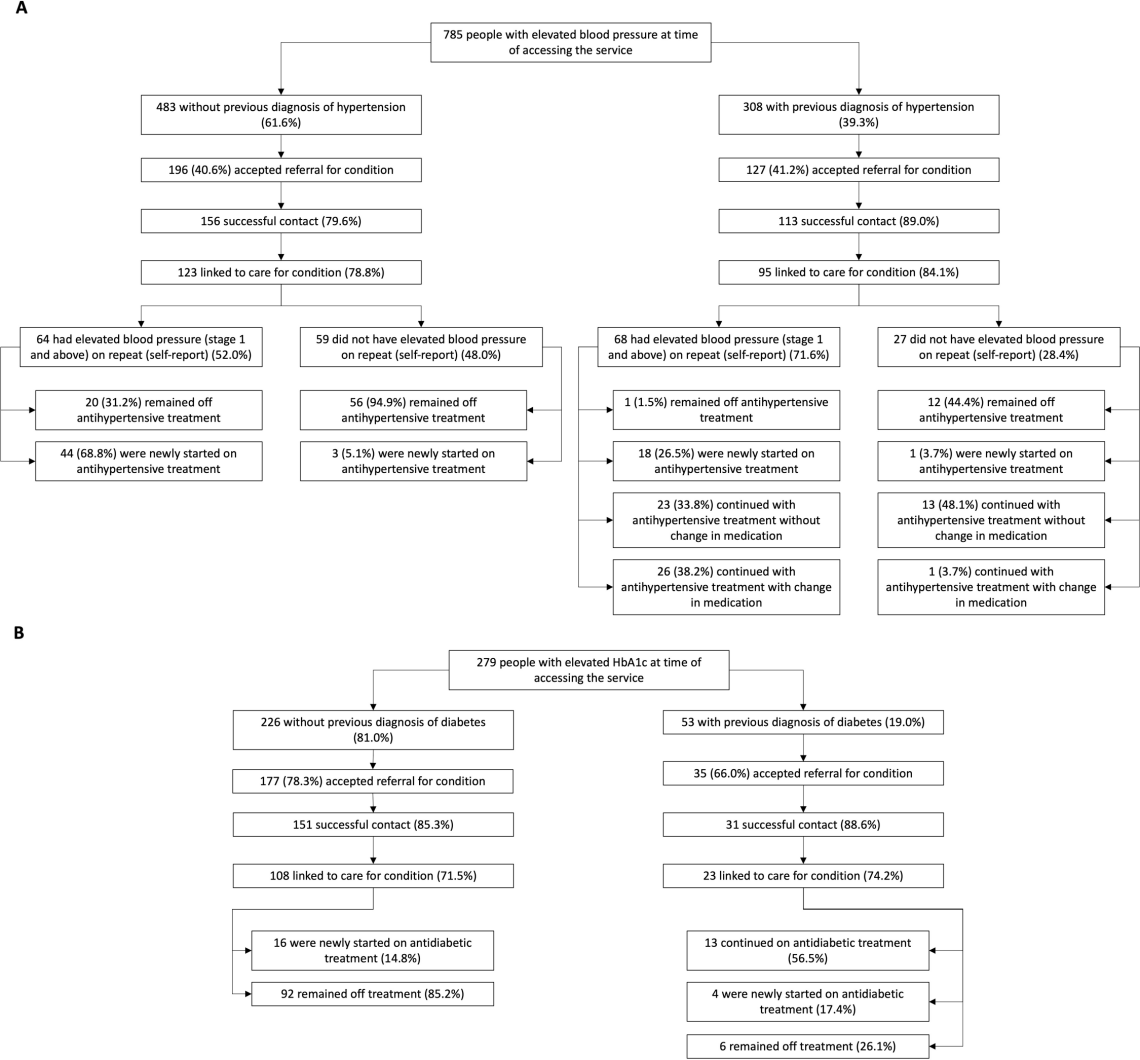
Flow diagram illustrating linkage to care after client referral among healthcare workers with an elevated blood pressure (A) or with an elevated HbA1c (B). A successful contact is defined as a participant having been reached telephonically and available for a follow-up telephonic conversation.

Less than half of the clients screening positive for hypertension (n=323/785, 41.1%) accepted referral. There was no difference in hypertension referral acceptance between those with previously diagnosed hypertension and those with no previous diagnosis (Fig 3A). Acceptance of referral for hypertension was more likely among older age-groups and those with higher body mass index (BMI; Table 1). There was no difference in referral uptake by HIV status.

Clients referred for a positive diabetes screen were more likely to accept referral (212/279, 72.0%) than those referred for hypertension (Fig 3B). Acceptance of referral for diabetes was more likely among older age-groups; but uptake was similar among people living with HIV, those testing HIV negative and those with unknown status (Table 1).

### Linkage to care

Among 323 people referred for hypertension, 269 (83.3%) were successfully followed up. Of those successfully contacted, 218/269 (81.0%) reported that they had linked to care for hypertension (Fig 1A, Fig 3A). Everybody who linked to care reported having had a repeat blood pressure reading: 64/123 (52.0%) of those without prior diagnosis of hypertension and 68/95 (71.6%) of those with known hypertension reported an elevated repeat blood pressure measurement. There was no difference in mean sBP or dBP at the time of screening between those who reported an elevated or a normal blood pressure at the repeat measurement (S1 Fig). A total of 111/132 (84.1%) clients referred and successfully contacted, with repeat elevated measurements, were started on antihypertensive treatment or had their antihypertensive treatment adjusted. For those who screened positive for hypertension, the highest attrition in the care cascade occurred at the step of acceptance of referral (462/785 [58.9%] did not accept referral; Fig 3A).

Of the 212 people referred for diabetes, 182 (85.8%) were successfully contacted and of those, 131 (72.0%) reported they had linked to care (Fig 3B). Among clients who linked to care, the proportion who had started or modified antidiabetic treatment at the time of follow-up differed by diagnosis (previously diagnosed or unknown) with 16/108 (14.8%) of those without a previous diagnosis of diabetes and 17/23 (73.9%) of those with a previous diagnosis of diabetes starting or modifying treatment.

Reasons for not linking to care were provided by all clients who reported not linking to care when they were initially contacted (n=112). The most common reasons given were work-related pressure (39/112, 34.8%) and insufficient time to follow up on their care (20/112, 17.9%). All clients said that they planned to link to care in the future. Repeat follow-up calls 3–6 months after the health check were successful for 45/112 (40.2%); the majority (35/45, 77.8%) of people reported that they had still not linked to care.

### Facilitators and barriers to linkage to care

**Fig 4:**
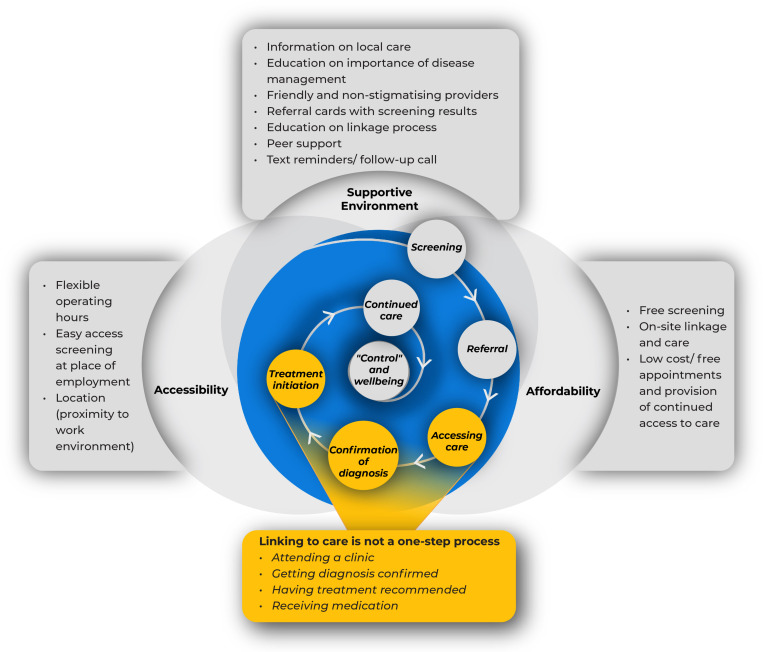
Conceptual framework of the facilitators in engagement with non-communicable chronic disease care.

A total of 19 in-depth interviews were conducted; 15 with clients receiving health checks and 4 with service providers who conducted health checks. The 15 clients recruited were from nine facilities, of which eight were male. Eleven screened positive for hypertension and four screened positive for diabetes. Eight had linked to care and the remaining had not accessed care, with most citing financial constraints as the biggest barrier. The interview duration ranged from 25-60 minutes. Thematic analysis identified 14 themes and highlighted several factors that impacted on clients linking to care (Table 2). The factors were categorised under i) a supportive environment, ii) accessibility and iii) affordability, all of which can act as facilitators or barriers to linkage to care. These were summarised and important strategies to address these barriers and developed into a conceptual framework (Fig 4). Table 2 highlights the key themes in detail including associated quotes.

**Table 2:** Quotations from in-depth interviews conducted with ICAROZ participants who linked to care (n=10) and who did not link to care (n=5) broken down into themes and codes.

Theme	Code	Quotation
Reasons for uptake of ICAROZ services	Pre-existing medical conditions	“Yes, I did but the time that my BP was high I was sick. And I just thought it was just because of me being sick at that time back in 2014…I never went to get my BP checked out again so this was an opportunity”. (Client, Male, 36)
		“It was poor management of BP that pushed me……I was diagnosed in 1998 and I was not taking my medication very well. I was not taking them as per prescribed combination.” (Client, Male, 52)
		“I was taking my medications as prescribed but maybe there was just something that had affected me at the time for the BP to be high.” (Client, Female, 42)
Facilitators for linking to further care	Comprehensive service provision	“You have the equipment with you…considering our health system. At times people might want to go and get treated…we don’t have glucose stripes. Which means one won’t be able to be tested for sugar. But as for you, you come with your own equipment and do supermarket approach.” (Client, Female, 30)
		“I would say it was quite an interesting experience. You know that moment when you get everything in one package. Getting your blood pressure checked, your eyesight, screened for COVID and even checked up on your mental health checked…having a one stop shop, it was a beautiful experience if I might say.” (Client, female, 31)
		“My temperature was checked, BP, height, weight and visual acuity and then a bit of some information on how to deal with stress management, and I also got an HIV test, my blood sugar was checked and also some health education on diet.” (Client, female, 30)
	Quality of care	“Those that I consulted would just say the BP is okay. But they would not explain clearly so much that I would be left hanging. So now that I heard that the program is back (ICAROZ) I am pleased and I actually came here running.” (Client, Male, 42)
		“What can I say for the quality of service? The service was good, it was professional. The ways they did their operations, everything was good, even when I was tested it didn’t take time, I arrived and got service immediately.” (Client, Female, 41)
	Accessible	“Then the other thing is that you are coming to us…at times we don’t create time to go for just a health check-up. We just then go for testing when we are already sick so I think it’s helping us a lot what you did that you came to us.” (Client, Female, 30)
	Risk and mitigation of livelihoods/uncertainty (family security)	“Personally, I am someone that values life. I look at the family that I have…my kids are still very young. Knowing that with BP you can just stroke, or just die whilst sitting down. So, I decided to take a step further so that I can work for my children, they are the ones who pushed me because they are still small…. even my parents they still want to be taken care of by me.” (Client, Male, 36)
		“As a father, and also looking at my family and knowing that diseases such as BP… You might just suddenly die… I was looking at the future of those that depend on me that if I suddenly die, then what will they do? So, it was my strong wish that I should be health.” (Client, Male, 44)
		“So, lack of interest and ignorance and not giving importance… but if you have children to look after you will seek for further care.” (Client, female, 42)
		“Realizing that if I’m to die because of BP, my family would lose its breadwinner, BP is a silent killer, it doesn’t have outstanding symptoms, my children are still young.” (Client, male, 42)
	Family medical history	“I realised from what my mother is going through, she also has BP, I decided that its better I start medication because it might be bad in the next years…I just decided to do the process because my mom since she was diagnosed, the BP is difficult to stabilise. It is not going down; she was actually referred to a physician.” (Client, Male, 36)
		“…like me I have a sister that died and we found out when she was about to die that she had diabetes. So, I started to think if I go (to the ICAROZ team) and they confirm it I might die if I don’t get on treatment.” (Client, Female, 46)
		“My family members, my brothers, my sisters, I
		told them, they accepted it because there is one of my brothers who is also leaving with that condition and is on medication, so they just said maybe it’s hereditary so don’t worry, it happens.” (Client, Male, 47)
	Family support	“My brothers were supportive they would say, “if you go to Karanda, with the amount of money that you have, you can get help.” So, they tried to give me assistance. They were saying the doctor’s consultation fee there is reasonable.” (Client, Male, 44)
	Accessibility	“We have a doctor here and usually staff will receive service free of charge in terms of consultation. Then for investigations and medications you use your medical aid card.” (Client, Female, 30)
		“It was not hard because I saw the doctor at my workplace. It’s where I work so there was no challenge that I faced. It was an advantage to me because there are no transport costs since things are harsh in the economy”. (Client, Female, 44)
		“The only doctor that comes does scans for pregnant mothers; he comes every Mondays. The rest we are supposed to go to Parirenyatwa, which is a bit far.” (Client, Female, 30)
		“We are consulted for free but as staff in terms of medication we are advised to use our medical aid.” (Client, Male, 44)
		“Here at the hospital, we have doctors so I saw one of them. That doctor is the one who wrote down that I have to do regular checks for BP and sugar (Diabetes).” (Client, Male, 57)
		“I was seen by doctor who works here. Every week, every day, we have a doctor.” (Client, Male, 35)
	Financial capacity	“On buying medication, its only at Premier (PSMAS) that they accept medical aid, otherwise we use cash, for me its affordable.” (Client, female, 44)
	Good quality and standard of care	“The place that I have talked about it might be a long distance to mission but the service is good. Everyone with different illnesses goes there, there is no one who went there and did not sing praises of them … “you will hear them say, I was almost dead. If it was not for that place, then I would have been dead.” (Client, Male, 44)
		“Because there was not a lot of people at the clinic so it didn’t take time to be served because I was one of the last people to go into the clinic because of work.” (Client, Male, 36)
	Access to medical aid	“I used HMMAS medical aid society It stands for Harare Municipality Medica Aid society. It covers all diseases …they deduct the money from our salaries…but for complicated procedures like scans and operations one would have to top up.” (Client, Male, 44)
		“To see the doctor, I didn’t pay anything because I used medical aid…then the medication I would pay $6 every month and to me its affordable.” (Client, Female, 44)
	Encouragement from ICAROZ Team	“The shortfall is not much; I think the only time I had a shortfall was at the eye section and it was a shortfall of about $18 US” (Client, female, 44)
		“What made me eventually link maybe the follow ups that you did Like what you did with, me you called me. I remember Molly calling and saying “sister what did you do after the screening?” I think that will make made me go even if I was relaxed.” (Client, Female, 30)
		“Yes, so it’s good what you are doing to do follow ups, at least it gives you time to think clearly as a person. That should I stay like this with the condition or should I fight for my health and you quickly do what is needed.” (Client, Female, 41)
Barriers to linkage to care	Poor service provision	“There are some hospitals that you know that if you go there, they will actually treat you roughly and so bad so much so that, even if you were sick, it becomes worse. When you arrive at the hospital you are made to sit on the benches expecting that the hospital will open at 8 and from 8am to 10am to even 11am and nothing has been done.” (Client, male, 35)
		“The hospital lacks a smooth flow. That make the patients return home with a happy face. “Every stage will have their fair share of attitude and pain that make you feel uncomfortable.” (Client, Male, 44)
		“…maybe at Parirenyatwa that’s where the doctor is available. But because you know how bad the service and care is at Parirenyatwa, you might choose not to go.” (Client, female, 30)
		**“…**you know that if you arrive at 6am you will leave the hospital at 4pm, that’s why someone might choose not to go where they have been referred to.” (Client, male, 35)
		“I know of one instance where this lady that I know was told that, you are only a staff member at your workplace and not here. So go in line and wait like the rest of the patients.” (Client, Male, 36)
	Financial barriers	“One might have been referred but the money to do further tests might not be available. Because you might arrive there and you are told of a huge amount and yet you don’t have the money.” (Client, Female, 44)
		“Considering our remuneration, you can be engaged and referred to go see a doctor but because of finance maybe you have no money for transport to go there.” (Client, Female, 30)
		“I was told that I should see a doctor for my eyes, but where do I get the money and where would I get the money for spectacles.?” (Client, female, 44)
		“So, I just thought I should stay like that, you see…the reason being that I cannot afford. Yes, I cannot afford, because of huge family responsibilities and it’s now a burden on my end, I have no solution.” (Client, male 61)
		“I was referred to get my eyes checked. Of which I wasn’t able to do it because of financial problems considering money and the money that we are paid looking at the situations in our families… and how expensive health is…that there is nothing you can do…there is no special treatment given to staff from health sector your earnings are supposed to assist you, but the earnings are not sufficient.” (Client, Male, 57)
		“That was my main issue because even to go to those good hospitals It would not be possible because at times there will be no money. So, we end up going to these places where they do not give you full diagnosis.” (Client, Male, 44)
		“Sometimes it’s because of economic status.
		That people concentrate on income generating issues. They always weigh on the benefits…” (Client, male, 52)
		“You see in our institutions. Like the issue of drugs. They don’t have, they will write you a prescription to go and buy in a pharmacy. The cost of the drug there. It’s expensive with these types of conditions we have people with conditions such as BP and they are stroking because of it… I couldn’t get nifedipine. At the same time, I didn’t have money to go and buy somewhere else so I just stopped taking them, so they will tell you the truth that I stopped taking it.” (Client, male, 57)
		“Mainly it’s the issue of money to buy the medication so, you buy and buy but when you are unable to buy, that’s when someone would want to stop medication. They stop taking the pills.” (Client, female, 44)
		“But the process for me to go and see the doctor had not yet been done. The money to cater for the expenses is unavailable. Things are not well for me. You might need to pay and also for other tests that you might be required to do.” (Client, Female, 41)
		“In our institutions on the issue on drugs they don’t have, they will write you a prescription to go and buy from the pharmacy. Then you will see that the drug that you need to buy it’s expensive. So, we have people with conditions such as BP who are stroking because of that. They will say, ‘I failed to get the drug I wanted, I couldn’t get nifedipine. At the same time, I didn’t have money to go and buy somewhere else so I just stopped taking’, so they will tell you the truth that I stopped taking it.” (Client, male, 61)
		“Yes, I have a plan to seek further care right now I don’t have a proper date to when I will go am still yet to decide when I can go. It’s just the issue of money.” (Client, Female, 41)
		“It’s costly, because I cannot pay $10 USD every month, it’s costly, yes, I could sacrifice but my family will suffer from hunger at home. I would cut down on children’s food.” (Client, Male, 40)
	Denial	**Based on age -** “Like at my age… at my age it’s impossible to be diagnosed with BP. I am 30, and at my clinic I actually told them I am still young, and if I start now being diagnosed with BP what more when I get old?”. (Client, Female, 30)
		**Based on Age** - “I got a little scared because honestly. I wasn’t prepared and it came to me as a shock and I was a little scared... Because thinking that I was diagnosed with diabetes at this age, ah no.” (Client, female, 30)
		“So, I have seen healthcare workers we tend to put a blind eye when it comes to our health and say we will see. Sometimes its fear, but sometimes is the lack of interest and ignorance. (Client, Female, 30)
		“What I have discovered, as healthcare workers we have a tendency to become reluctant when it comes to things that concerns our health, that’s where I think there is an issue.” (Client, Male, 57)
		“Someone would have not accepted the diagnosed condition that it is possible in such situation. Acceptance is the problem is.” (Client, Male, 35)
		“Sometimes they might diagnose the condition and you are not fully convinced and you haven’t accepted. At times you would have doubt that even taking the medication you will not do it properly. So, there is need for time to digest then you go when you are ready and even if they put you on medication.” (Client, Female, 41)
	Lack of Knowledge	“BP is a condition which requires that when it is diagnosed you get the treatment and counselling. So, if you don’t have such information, you will think I’m not the only one, so let me just let it be.” (Client, Male, 57)
		“Some people might not have the full information on what harm the condition can cause. Or even just ignorance of not acknowledging that they should go for consultation at the doctor.” (Client, Male, 36)
	Hesitation based on other people’s experiences-	“I’m just hesitant…because my mother is also on medication, I feel like it’s a burden, it troubles her a lot. When she started, she was taking HTC? But regularly she would change the medication and up to now she is still being changed. So, I see as if it’s a little bit burdening. So, she will just be changing so I felt like maybe to maintain it on my own is better.” (Client, Female, 30)
		“But for me personally, hearing from other people who have those conditions, I just thought I could manage.” (Client, Male, 47)
	Pill burden	“Well one reason is that when you start taking the medication it becomes difficult to stop when you want to so you would have messed up your system…one would want to stop because they no longer have money to buy medication.” (Client, female, 44)
		**“**Well, if you take BP pills you won’t be able to control it naturally, it would just require pills all the time…I saw it happening because my mom can’t go without the pills, she will be weak and almost collapsing with severe headache” (Client, Female, 30)
		“Another thing is that, pill burden is annoying.” (Client, Female, 46**)**
		“For me I had fear to go to the doctor and get pills because the medication is a life time pill, so you might fail to maintain it plus it’s expensive to get or buy. So I was afraid that…. If I am put on BP treatment, I might fail to buy the pills.” (Client, Female, 42)
	Fear and self-negligence	“People have fear of starting medication. That is what I have discovered in my line of service. People are afraid of discovering new diseases on them…they fear that if they discover another disease on me, how will I manage or how will I deal with it.” (Client, Female, 30)
		“I had a high BP reading for some time and I let it go because I was scared…sacred of medication, so I let it go and even ignored the check-ups. I actually realised that it might actually go higher so let me stop the check-ups.” (Client, Female, 30)
		“A layman who is coming from home will listen more than someone who is in healthcare already and that’s true from what I have seen.” (Client, female 30)
		“So, I have seen as healthcare workers we tend to put a blind eye when it comes to our health and say we will see. Sometimes its fear, but sometimes is the lack of interest and ignorance.” (Client, Female, 30)
		“What I have discovered, as healthcare workers we have a tendency to become reluctant when it comes to things that concerns our health, that’s where I think the issue is” (Client, Male, 57)
		“Some don’t have health priorities. So, they might think it’s a waste of time. People feel like they are okay until they become worse somehow. Sometimes people just make a self-diagnosis that all is well, yet its carelessness.” (Client, Male, 52)
		“Then we can also say fear of the unknown that if I go, they will say its diabetes what then will I do maybe they say its HIV what then will I do. Those are some of the fears that will be there.
		Fears to handle the results.” (Client, Male, 47)
	Low risk perception	**BP Asymptomatic – “**If we look at BP, someone can say I don’t feel anything and just say I will see it later on and ignore the issue.” (Client, Male, 44)
		“If they had asked me what test I need done, I would not have been tested for BP because I was not feeling anything at that time.” (Client, male, 57)
		“I used to take it light until I was taken to Gomo (Central hospital) in an ambulance. It wasn’t because I was down. I was not feeling anything but the BP reading was too high…that was in 2014.” (Client, Male, 52)
	Lack of time	“You will be referred to go somewhere and right now you know our work is hard, we are short-staffed. You cannot say I can’t come to work; I am at a queue to see the doctor; you will see it better not to go… they are slow to serve you. And the time that the doctor comes you would be supposed to be at work. So, you end up going back before the doctor consults.”(Client, Female, 44)
		“So, the time that I will waste standing in a queue especially at the general healthcare facilities. I decide I would rather let it be.” (Client, Female, 30)
		“Sometimes you might finish late at work and you might think twice to go and see a doctor after work. Then you would then have to wait for off days. But maybe during this time of waiting the condition is worsening and damaging.” (Client, Male, 36)
		“I don’t want to lie to you, people are very busy these days you can refer someone here and when they go back home, they forget everything.” (Client, Female, 30)
		“If I don’t come to work for a day, that day will be removed from my salary because I am paid according to hours worked. And going to a public health institution there is no way I will receive service on a weekend.” (Client, Female, 46)
		“We might say because of work time and schedule.…you might be needed at work on time. So going from work to the other place it’s something that is impossible because you will be required at work every day from 8am to 5pm and also the service that you were referred to might not also open on weekends.” (Client, Female, 42)
	Perception of sickness as non-medical	“There are those certain diseases that are said to be spiritual, like BP, so some might think going to the hospital doesn’t work” (Client, Female, 44)
		“Where they will say we have prayed for you with water or even these Pentecostal now which claim that we have prayed for you so you are delivered so what can you do. They will pray for you.” (Client, Female, 42)
		“There are some healthcare workers who did not get the vaccine claiming that they are not allowed to do so at church, so the same goes with medication for things like BP as well…” (Client, Male, 40)
	Restrictive medical aid	“So, it depends with the institution that you would have gone to but most do not cover specialist care like eyes. So, you would have to pay extra money.” (Client, Female, 44)
		“I thought I would be using my medical aid but I do not know what happens with these medical aids. I feel like if am paying medical aid, it should be able to cover for the medication and the processes I want. So, for you to now top up using USD and of which you are not even paid using USD. It becomes a challenge now. the place I went to buy BP medication did not accept swipe.” (Client, male, 57)
		“It covers the consultations and when dealing with private clinics, some want the medical aid and some will not accept it. Most of the time you will hear that it is not accepted in most clinics …its HMMAS from work.” (Client, Male, 36)
	Alternatives to medical care	**Herbs** “You find that most of the people including healthcare workers, they believe in herbs and these herbs are too many…you find that among healthcare workers. We still have a number that believes that we can rely on herbs. So, there is a tendency that someone might drop medication and try to use the herbs. The percentage is high.” (Client, Male, 35)
		**Herbs** - “People have different beliefs. If they believe in herbs and other stuff, they will decide to go there then forgo going to the doctor. There are some I heard saying about moringa for BP treatment.” (Client, Male, 44)
		**Herbs** - “Zumbani and other stuff. If you take a potato and put it in water and drink the water. The condition might get better. They say it lowers BP.” (Client, Female, 41)
		**Herbs**…” Especially those ones getting advertised these days. Where someone will come and say if you use this herb. It will reduce BP; it will reduce sugar and all the chronic problems. We hear of people coming saying this one helps on BP. Let me just look for the cheap stuff.” (Client, male, 61)
		**Herbs** - “And using these herbs like if you eat blackjack…If you drink avocado leaves that is what I do sometimes when am at home. I have blackjack, I boil them and drink the water and also the avocado leaves. That is what I usually use because that is what I am able to get. I also use ginger, not every day, twice or three times a week.” (Client, Female, 42**)**
		**Herbs- “**Then also others advised me to use herbs I am using avocado leaves, then am drinking lemon water every day in the morning. Yah, I feel some changes because in the past I would feel weak and dizzy because of diabetes, just an unexpected dizziness. But now I no longer have that.” (Client, Male, 47
		**Herbs**- “Actually I compared the two conditions then I saw that there are herbs that can assist for sugar(diabetes) and contain it so for the meantime, I will contain diabetes and see.” (Client, Male, 47)
		**Traditional medicine-** “Some believe in the throwing of spiritual stones and bones or even eat raw roots from the bush. Of which the medicinal content in those roots. Might be far beyond the required limits by the hospital. So, you might actually over dose even though it’s the right medicine, which can be harmful if you overdose.” (Client, Male, 44)
	Opting for lifestyle adjustment	**“**So, when I got home it was an issue of limiting things like those from my diet, it looks like it’s helping bit by bit, though I’m not on medication” (Client, male, 57)
		“They maintain it by trying not to think a lot.” (Client, female, 42)
		“On diabetes...I have not yet been linked. I think am managing, I am trying to control with my diet, I was thinking that if it fails to be controlled by diet… I have limited the amount of Sadza and I do exercise every day in the morning, I run.” (Client, Male, 47)
	Invasion of privacy worries	“Maybe they might feel like invasion of privacy if they are to go and tell the doctor about their condition. So that might make someone not want to link up. Invasion of privacy.” (Client, female, 30
Reasons for self-selecting service providers	Quality and standard of care	“Someone might just be happy to be referred to a clinic but the clinic that they go might have low standards. They cannot even explain clearly to the patient so that the patient comes out of the clinic fully satisfied.” (Client, Male, 44)
		“I remember I went to Sekuru Kaguvi where you had referred me to in November and I was booked to be seen on 14 February. So, I saw that the time I was to wait was too long…what I was feeling at the time, I was struggling with my eyesight. I wanted to be attended urgently. I changed and I went for a private doctor.” (Client, Male, 35)
		“The place that I have talked about it might be a long distance to mission but the service is good. Everyone with different illnesses goes there, there is no one who went there and did not sing praises of them … you will hear them say, ‘I was almost dead. If it was not for that place, then I would have been dead’.” (Client, Male, 44)
	Affordability	“I choose to see a doctor that is here at the hospital rather than going to see a private one. It will be a bit cheaper for me. But that doctor will not give me detailed advice. They will just rush through and take the consultation for granted because they think I know how I am supposed to help myself.”
	Efficiency	“I went to a private clinic called Nkwisi gardens in Westlea, its faster there than government institutions.” (Client, Male, 36)
	Convenience	“Maybe a person would just decide let me just go straight to a pharmacy and tell them am sick of this and then buy the medication and go.” (Client, Male, 36)
		“I would go and say I want to know my BP reading. Some pharmacies would charge $2 for a single BP check.” (Client, Male, 44)

### Facilitators to uptake of further care

In the interviews, when clients were asked why they linked to care, some highlighted that accessibility and the facility’s reputation in terms of quality of care and service provision were key contributing factors. An accessible facility meant that distance and transport costs were negligible, therefore it was convenient for clients to link to care without worrying about time and costs. However, if health services and providers had a good reputation, clients said they would happily access care there even if they incurred transport costs and/or had to spend time to get to a facility because they felt that they would get good quality care.

“It was not hard because I saw the doctor at my workplace. It’s where I work so there was no challenge that I faced. It was an advantage to me because there are no transport costs since things are harsh in the economy.” (Client, Female, 44)

“The place that I have talked about, it might be a long distance to get there… but the service is good. Everyone with different illnesses goes there, there is no one who went there and did not sing praises of them … You will hear them say, ‘I was almost dead. If it was not for that place, then I would have been dead’.” (Client, Male, 44)

The perception of risk was a facilitator to linkage influenced by the client’s personal experiences. Family medical history, or the death of a loved one, and understanding the morbidity and mortality associated with untreated chronic conditions was a strong motivator for some clients to link to care:

*“… I have a sister that died, and we found out when she was about to die that she had diabetes. So, I started to think if I go (to access the service) and they confirm it… I might die if I don’t get on treatment.” (Client, Female, 46)*

*“I realised from what my mother is going through, she also has BP, I decided that its better I start medication because it might be bad in the next years…I just decided to do the process because for my mom - since she was diagnosed - the BP has been difficult to stabilise. It is not going down; she was actually referred to a physician.” (Client, Male, 36)*

*“As a father, and also looking at my family and knowing that diseases such as BP… You might just suddenly die… I was looking at the future of those that depend on me that if I suddenly die, then what will they do? So, it was my strong wish that I should be healthy.” (Client, Male, 44)*

Support from family, peers, or service providers was reported as an important factor in facilitating continuous care. A client reported that “*My brothers were supportive they would say, ‘if you go to (Facility C), with the amount of money that you have, you can get help.’ So, they tried to give me assistance. They were saying the doctor’s consultation fee there is reasonable*.” In addition, one client felt that the follow-up calls by the team who provided the health checks supported her in seeking care.

Access to a comprehensive medical aid package facilitated linkage to care, as clients who could afford to register on such medical aid schemes reported experiencing no or limited out-of-pocket costs when seeking further care:

*“To see the doctor, I didn’t pay anything because I used medical aid… On buying medication, it’s only at medical Facility R that they accept medical aid, otherwise we use cash, for me its affordable.’’ (Client, Female, 44)*

### Barriers of linkage to care

Findings from the in-depth interviews highlighted some of the barriers clients were experiencing when trying or considering to link to further care. Perceived low quality services provided at certain health facilities was cited as a major deterring factor:

“…maybe at Facility C that’s where the doctor is available… but because you know how bad the service and care is there you might choose not to go.” (Client, Female, 30)

Perception of risk was not just as a facilitator but also as a barrier to continuous care. It was largely influenced by perceived association between personal attributes and risk of chronic conditions. Clients who were younger or did not have any symptoms perceived themselves as unlikely to have a chronic condition which needed treatment, making them less likely to seek care. Some clients were in denial of the health problem identified through the health check and did not think it was necessary to link to care.

*“Like at my age… at my age it’s impossible to be diagnosed with blood pressure. I am 30, and at my clinic I actually told them I am still young, and if I start now being diagnosed with blood pressure what more when I get old?”. (Client, Female, 30)*

*“If they had asked me what test I need done, I would not have been tested for blood pressure because I was not feeling anything at that time.” (Client, Male, 57)*

Fear was another major barrier for clients in seeking treatment. Some of the denial or the acknowledgment that there was a problem emanated from fear of being on lifelong medication. Clients were also afraid of side effects and concerned that they may have to repeatedly change medication to find a drug which worked for them.

*“I’m just hesitant…because my mother is also on medication, I feel like it’s a burden, it troubles her a lot… I had a high BP reading for some time and I let it go because I was scared…scared of medication, so I let it go and even ignored the check-ups. I actually realised that it might actually go higher so let me stop the check-ups.” (Client, Female, 30)*

*“For me I was afraid to go to the doctor and get pills because the medication is a lifetime pill, so you might fail to maintain it plus it’s expensive to get or buy. So I was afraid that…. If I am put on BP treatment, I might fail to buy the pills.” (Client, Female, 42)*

Health check service providers also pointed out that perception of risk and fear of medication was among the main reasons for clients not to accept referrals.

*“Most clients were refusing referrals with some saying, “Don’t worry we can do daily checks for BP, diabetes…” (…). The other reason for refusing referral was that they did not want to be initiated on antihypertensive drugs, and diabetic drugs. Some indicated they would agree to the referral only when their BP or glucose levels are high but for now, they are not ready to start on medication which is taken daily for life.” (Health Check Nurse, Female)*

Clients reported “not linking to care” because they chose to change their lifestyle or accessed alternatives to “modern Western” medicine, for example herbs.

*“People have different beliefs. If they believe in herbs and other stuff, they will decide to go there then forgo going to the doctor. There are some I heard saying about moringa for BP treatment.” (Client, Male, 44)*

*“So, when I got home it was an issue of limiting things like those from my diet, it looks like it’s helping bit by bit, though I’m not on medication” (Client, Male, 57)*

Financial constraints were a major barrier repeatedly highlighted. Poor remuneration, compounded by unaffordable direct costs (clinic user fees, diagnostics, and treatment) and indirect costs (transport) were some of the cited barriers.

*“Considering our remuneration, you can be engaged and referred to go see a doctor but because of finance maybe you have no money for transport to go there.” (Client, Female, 30)*

*“I was referred to get my eyes checked. Of which I wasn’t able to do it because of financial problems considering money and the money that we are paid looking at the situations in our families… and how expensive health is…that there is nothing you can do…there is no special treatment given to staff from health sector your earnings are supposed to assist you, but the earnings are not sufficient.” (Client, Male, 57)*

Several clients mentioned that healthcare workers working in the public sector were entitled to medical aid subsidies by the government. If healthcare workers decided to sign up to the medical aid package, their contribution was directly deducted from their salaries. However, contrary to the comprehensive medical aid schemes (which one would individually pay for), those subscribing to the medical aid subsidies by the government complained that it was a low-ranking package, with restricted coverage. This meant that they had limited access to consultations free-of-charge or at reduced costs, and access to only basic drugs. Most of the clients interviewed had encountered shortfalls themselves or knew of colleagues who had not been able to access care, or had to pay extra, despite having medical aid (64.2% of clients reported having medical aid at baseline). Some clients explained that because of the shortfalls, they had decided against the medical aid offered by the government and instead self-funded a better, but more expensive, medical aid package. A client provided some detailed insights about the medical aid scheme provided as staff benefits, detailing what was and was not affordable:

*“So, it depends on the institution that you would have gone to, but most (government funded medical aid schemes) do not cover specialist services like eye-care. So, you would have to pay extra money...” (Client, Female, 44)*

*“You see in our institutions. Like the issue of drugs. They don’t have, they will write you a prescription to go and buy in a pharmacy. The cost of the drug there. It’s expensive with these types of conditions we have people with conditions such as BP and they are stroking because of it… I couldn’t get nifedipine. At the same time, I didn’t have money to go and buy somewhere else so I just stopped taking them, so they will tell you the truth that I stopped taking it.” (Client, Male, 57)*

## Discussion

In this study, we found that 81% and 72% of those screening positive for hypertension and diabetes, respectively, had linked to care within 30–60 days of screening, which is on the higher end of linkage rates found in other studies in LMICs (49–83%) [29–32]. Of note, studies with higher linkage rates provided transport vouchers and targeted health education to facilitate the process. In our study, which exclusively focused on health workers, it was anticipated that barriers to linkage such as access, costs, and lack of awareness of the potential implications of not getting treatment for a chronic condition would be easier to overcome. These were however still the main barriers experienced. Given the high unemployment rates in Zimbabwe the general population is likely to experience even more severe challenges in completing the care cascade than the healthcare workers enrolled in the study.

Contrary to other studies, we conceptualised the linkage process in two steps: step one “accepting referral” and step two “seeking onward care”. The rationale for differentiating between the acceptance of referrals and seeking care was that our target population, health workers, was likely to have a higher health-literacy than the general population. Hence, we hypothesised that health workers may feel more empowered to make an informed decision about their own health and decide not to take up a referral. This is in contrast to the general population which may not want to challenge or question what they are told by healthcare providers. They may accept a referral despite knowing that their circumstances will not allow them to link to care resulting in what is often termed “silent refusals” (for example people accepting referral, but not seeking onward care) [33]. Our service providers were specifically trained in providing information and education and respecting people’s autonomy [6]. While every client who had an abnormal result was supposed to be offered a referral, providers were aware that they were dealing with their peers and accepted when a client decided not to take up the referral. The power imbalance which typically exists between a healthcare provider and client was thus likely to be less marked due to the clients and providers being peers, and the training received by providers. Additionally, service providers experience similar workplace and economic challenges as their clients and were thus aware of the challenging circumstances which act as barriers to accessing care. We suggest, therefore, that only clients who actually planned to link and had a high likelihood to making it happen, took up referral. This in turn means that the reported linkage rates in this study may be higher compared to other studies where there are “silent” refusals.

Given the severe economic instability Zimbabwe has experienced over the last two decades it is not surprising that financial constraints were frequently reported as a major barrier to linkage to care [34]. This has also been reported by other studies conducted in Uganda, Kenya, and Malawi [29,31,35,36]. Research suggests that integrated screening programs for NCDs, particularly in low-income settings such as Zimbabwe, can offer substantial advantages in terms of cost [37–41]. A systematic review found that integrated screening for diabetes and cardiovascular diseases in low-resource settings was generally cost-effective, [41] though this was context dependent: screening programs that do not ensure effective linkage and treatment initiation/retention may lead to wasted resources as the benefits of early detection are lost without proper management of the conditions identified. While there is an upfront cost, providing the infrastructure for appropriate downstream processes after screening increases the likelihood of positive health outcomes, thereby maximizing the return on investment for screening activities.

In the Zimbabwean economic context, financial incentives to attend follow-up appointments would very likely have increased linkage to care, as shown in a study conducted in Kenya and Uganda [42]. However, linkage to care is just one step in the care cascade and does not guarantee continuous engagement. This is especially pertinent in the context of NCDs which often require confirmatory testing, further diagnostic work-up, and combinations of lifestyle changes and long-term adherence to optimised medication. Current health systems in LMICs are not configured to support people with NCDs whilst services are typically secondary-care based and siloed, with multiple locations and providers of care being particularly ill-suited to the needs of people with more than one health condition (multimorbidity) [43]. Follow-up calls were perceived by clients in this study as “caring and supportive” and have been shown to improve linkage to care in the context of HIV [44]. However, such interventions will be insufficient without addressing system challenges, including ensuring accessible, joined-up and affordable (or ideally free) NCD services and medications.

The results of our study clearly show that diagnosing a condition does not automatically translate into disease control and thereby prevention of morbidity and mortality. A high proportion of clients previously diagnosed with hypertension and diabetes were not receiving treatment and/or their condition was not well controlled when they attended the health check. Whilst a person may not immediately link to care after diagnosis, undergoing screening, receiving actionable results combined with health education may facilitate linkage to care at a later stage or incite lifestyle changes. Most clients who screened positive for hypertension and/or diabetes were also overweight or obese, indicating that changes in lifestyle may prevent development of NCDs and/or improve control. Service providers often focus on diagnosis, linkage, and adherence because these are easily measurable programme indicators. However, the ultimate aim of NCD programmes should be reduction of long-term morbidity and mortality. Linkage to care is part of the process, ideally leading to disease control and aversion of complications, and needs to be patient-centred to be effective. The overarching themes identified in this study (accessibility, affordability, and support) are likely to impact not just on linkage, but the entire care cascade.

Lessons can be drawn from HIV care, which has seen great progress due to adequate funding and implementation of decentralized care, demonstrating that long-term care for chronic conditions is achievable at scale [45,46]. Further, integration of additional services (including NCD care) into HIV care as part of differentiated service delivery have shown the benefits of more person-centred models of care to improve patient outcomes whilst being cost-effective [47]. However, provision of NCD/HIV care for all who need it remains incomplete, whilst NCD services, for people who do not have HIV lag even further behind. The principles and benefits of differentiated service delivery, which include a prioritisation on person-centred care and accessibility, should continue to be scaled up and scaled out to include the wider population. This should be complemented by public awareness and education campaigns, including myth-busting, to increase participation in NCD testing and treatment, including reducing fear of medication and side effects. In Zimbabwe and similar settings in southern Africa, people with NCDs face clinic fees, medication costs, and transport barriers, unlike free HIV services.

Strengths of this study include the large sample size and the use of a mixed-method approach. The study recruited all cadres of healthcare workers across seven of the ten provinces of Zimbabwe and from different levels of facilities. As such, the results are likely to be generalisable to similar settings in neighbouring countries. Furthermore, considerable efforts were made to contact clients to assess linkage to care: multiple phone numbers were recorded, these were confirmed by a short call after the visit, and there were multiple attempts to contact people for follow-up.

The study has several limitations. Some clients could not be reached during follow-up, which may have introduced selection bias as it is possible that people who were more difficult to contact were less engaged or had more competing demands, and therefore were less likely to have linked than those who were contacted. Linkage to care, repeat blood pressure measurement, and initiation or change of treatment were self-reported and hence subject to both social desirability and recall bias. Lastly, both hypertension and diabetes are conditions which require repeated measurements and assessments before a final diagnosis is made. As our screening was based on testing on a single day, we were unable to make a definitive diagnosis of hypertension or diabetes and therefore had to define clients as having screened positive for hypertension and/or diabetes.

## Conclusions

In the context of epidemiological transition and the rapidly increasing prevalence of NCDs in LMICs, we clearly need to pay more attention to diagnosis, care, and prevention of these conditions. Here we show that healthcare workers, who are arguably more educated and have better health literacy than the general population, struggle to complete the early steps in the continuum of care. There is an urgent need to design person-centred programmes to cater for NCDs to prevent the looming epidemics of stroke, cardiovascular disease, chronic kidney disease, and diabetic sequelae such as neuropathy and retinopathy. Interventions must address systemic challenges and ensure a sustainable, accessible, affordable, and supportive service throughout the NCD care cascade.

## Supporting information

S1 Fig Distribution of hypertension blood pressure measurements stratified by whether the reported repeat measurement was elevated or not. (DOCX)

S1 Table Characteristics of clients recruited for in-depth interviews. (DOCX)

S1 Checklist Inclusivity in global research. (DOCX)
