# Supplementary material for: Barriers to and facilitators of linkage to care following hypertension and diabetes screening among health workers in Zimbabwe: A mixed method study
Source: PLOS Glob Public Health. 2025 Apr 29;5(4):e0004513. doi: 10.1371/journal.pgph.0004513 (PMC12040240; doi:10.1371/journal.pgph.0004513)
Supplement: S1 Fig — (DOCX) [file pgph.0004513.s001.docx]

**
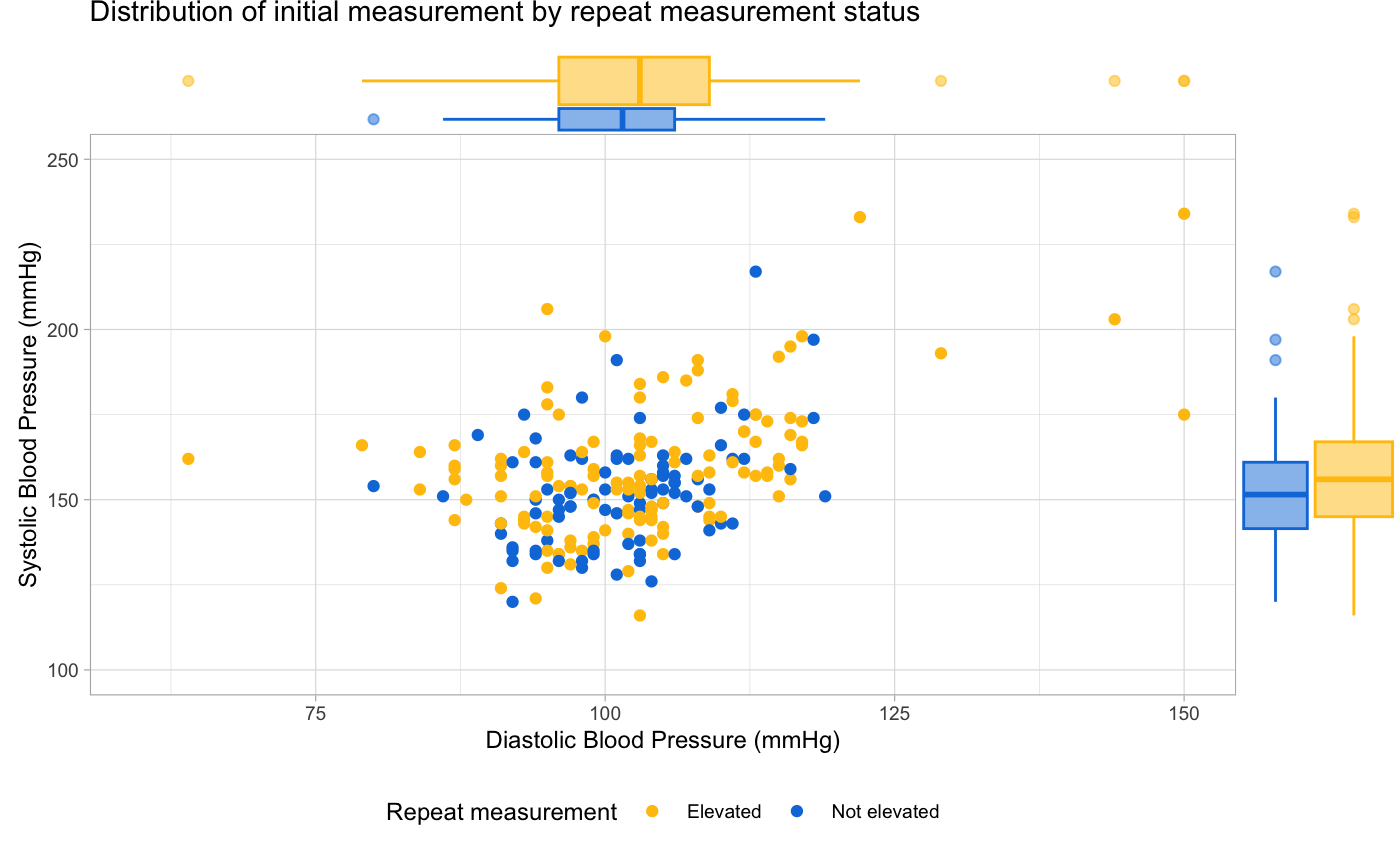
**

**S1 Fig: Distribution of hypertension blood pressure measurements stratified by whether the reported repeat measurement was elevated or not**.

***A****. Scatterplot of clients with an elevated blood pressure stratified by whether the reported repeat measurement was elevated (blue: not elevated, yellow: elevated). Colour-coordinated boxplots show the median with associated IQR of the sBP (vertical) and the dBP (horizontal).*
