# Supplementary material for: Barriers to and facilitators of linkage to care following hypertension and diabetes screening among health workers in Zimbabwe: A mixed method study
Source: PLOS Glob Public Health. 2025 Apr 29;5(4):e0004513. doi: 10.1371/journal.pgph.0004513 (PMC12040240; doi:10.1371/journal.pgph.0004513)
Supplement: S1 Table — (DOCX) [file pgph.0004513.s002.docx]

**S1 Table: Characteristics of clients recruited for in-depth interviews**

*15 people recruited from nine facilities. Eight were male, and seven were female. The majority had high blood pressure results, and some had comorbidities. Ten had linked to care and remaining had not accessed care, with most citing financial constraints as the biggest barrier.*

| **Site** | **Sex** | **Age** | **Condition** | **Linked to care** | **Reasons for not linking to care** |
| --- | --- | --- | --- | --- | --- |
| 1 | M | 35 | Hypertension | No | Financial constraints |
| 2 | F | 44 | Hypertension | Yes |  |
| 3 | F | 30 | Hypertension | No | Financial constraints |
| 3 | M | 36 | Hypertension | Yes |  |
| 4 | F | 30 | Diabetes | Yes |  |
| 4 | M | 57 | Hypertension and diabetes | Yes |  |
| 5 | M | 35 | Hypertension | Yes |  |
| 5 | F | 46 | Diabetes | Yes |  |
| 6 | M | 52 | Hypertension | Yes |  |
| 3 | F | 42 | Hypertension | Yes |  |
| 7 | F | 41 | Hypertension and vision | Yes (for vision) |  |
| 7 | F | 41 | Diabetes | No | Financial constraints |
| 8 | M | 47 | Diabetes and vision | No diabetes, yes vision | Not stated |
| 9 | M | 40 | Vision, hypertension, diabetes | No for vision, yes for other conditions | Distance, time, financial constraints |
| 9 | M | 61 | Hypertension and vision | Yes |  |
